# Identification of Morphogenesis-Related NDR Kinase Signaling Network and Its Regulation on Cold Tolerance in Maize

**DOI:** 10.3390/plants12203639

**Published:** 2023-10-21

**Authors:** Ran Tian, Sidi Xie, Junjie Zhang, Hanmei Liu, Yangping Li, Yufeng Hu, Yubi Huang, Yinghong Liu

**Affiliations:** 1 State Key Laboratory of Crop Gene Exploration and Utilization in Southwest China, Sichuan Agricultural University, Chengdu 611130, China; tianranchn@163.com (R.T.); xiesidichn@163.com (S.X.); liyangping163@163.com (Y.L.); huyufeng@sohu.com (Y.H.); 2 College of Life Science, Sichuan Agricultural University, Ya’an 625014, China; junjiezh@163.com (J.Z.); hanmeil@163.com (H.L.); 3 Maize Research Institute, Sichuan Agricultural University, Chengdu 611130, China

**Keywords:** maize, MOR signaling network, cold stress, cold tolerance

## Abstract

The MOR (Morphogenesis-related NDR kinase) signaling network, initially identified in yeast, exhibits evolutionary conservation across eukaryotes and plays indispensable roles in the normal growth and development of these organisms. However, the functional role of this network and its associated genes in maize (*Zea mays*) has remained elusive until now. In this study, we identified a total of 19 maize MOR signaling network genes, and subsequent co-expression analysis revealed that 12 of these genes exhibited stronger associations with each other, suggesting their potential collective regulation of maize growth and development. Further analysis revealed significant co-expression between genes involved in the MOR signaling network and several genes related to cold tolerance. All MOR signaling network genes exhibited significant co-expression with *COLD1* (*Chilling tolerance divergence1*), a pivotal gene involved in the perception of cold stimuli, suggesting that COLD1 may directly transmit cold stress signals to MOR signaling network genes subsequent to the detection of a cold stimulus. The findings indicated that the MOR signaling network may play a crucial role in modulating cold tolerance in maize by establishing an intricate relationship with key cold tolerance genes, such as *COLD1*. Under low-temperature stress, the expression levels of certain MOR signaling network genes were influenced, with a significant up-regulation observed in *Zm00001d010720* and a notable down-regulation observed in *Zm00001d049496*, indicating that cold stress regulated the MOR signaling network. We identified and analyzed a mutant of *Zm00001d010720*, which showed a higher sensitivity to cold stress, thereby implicating its involvement in the regulation of cold stress in maize. These findings suggested that the relevant components of the MOR signaling network are also conserved in maize and this signaling network plays a vital role in modulating the cold tolerance of maize. This study offered valuable genetic resources for enhancing the cold tolerance of maize.

## 1. Introduction

The MOR signaling network is indispensable for the proper growth and development of eukaryotes [1,2]. The signaling pathway, initially discovered in yeast, plays a crucial role in governing cell morphogenesis, polarity, and cytokinesis in *Saccharomyces cerevisiae* [3,4,5,6,7,8,9]. Mutations in the MOR components of yeast result in cellular demise or impairments in cellular polarity [6,10]. The MOR signaling network is highly conserved across eukaryotes and likely plays a pivotal role in the regulation of plant stem cell maintenance as well as cell polarization [1,2].

The MOR signaling network comprises two protein kinases, KIC1 (Kinase that interacts with cell division cycle1) and CBK1 (Cell wall biosynthesis kinase1), along with three associated proteins, MOB (Mps one binder), MO25 (Mouse embryo scaffolding protein25) and TAO3/FRY (Transcriptional activator of OCH1/Furry) [1,6,11,12]. Among them, CBK1 serves as the pivotal component of this regulatory pathway. This protein is a serine-threonine protein kinase belonging to the NDR (Nuclear Dbf2 Related) kinase family and exerts its influence on cellular growth and development through phosphorylation of downstream effector proteins [1,2,13]. The activation of CBK1 is facilitated by MOB, and its interaction with CBK1 is essential for the regulation of kinase activity [6,8]. KIC1 is a GCK (germinal center kinase) in the Ste20 (sterile 20 protein) kinase family and is a MAP4K (mitogen-activated protein kinase kinase kinase kinase) which phosphorylates CBK1, thereby activating its function [12,14]. The protein MO25 functions as an activator for KIC1, while TAO3/FRY serves as a mediator linking the interaction between KIC1-MO25 and CBK1-MOB, thereby facilitating the phosphorylation of CBK1 by KIC1 [9,12,15].

Cold stress significantly impedes the growth, development, and distribution of plants, thus, posing a substantial threat to agricultural production [16]. Plants have developed diverse molecular mechanisms to effectively respond to cold stress, and several key regulatory factors in response to this environmental condition have been identified [17,18,19,20]. The perception of cold stimuli involves the cell membranes, calcium channels, and COLD1, a gene encoding G-protein regulator [18,19,21]. After perceiving the cold stimulus, plants initiate a diverse array of regulatory networks to induce the expression of *COR* (*Cold regulated*) genes [19]. The CBF/DREB1 (C-repeat binding factor/Dehydration-responsive element binding factor1)-dependent transcriptional regulatory pathway represents the central mechanism underlying plant responses to cold stress [22]. The up-regulation of the CBF/DREB1 gene in response to low temperature triggers the activation of the COR gene’s expression and subsequent accumulation of protective substances, including osmolytes and cryoprotective proteins, thus enhancing cold tolerance [22]. ICE1 (Inducer of CBF expression1) and ICE2 facilitate the expression of CBF/DREB1 genes, thereby positively regulating cold tolerance [23,24,25]. The phosphorylation of ICE1 is a critical factor in the regulation of plant cold tolerance, and under cold stress conditions, the *Arabidopsis* protein kinase OST1/SnRK2.6 (Open stomata1/SNF1-related protein kinase2.6) phosphorylates ICE1 to enhance its transcriptional activity and stability [26]. Moreover, *Arabidopsis* MPK3 (mitogen-activated protein kinase3) and MPK6 phosphorylate ICE1, thereby attenuating its protein stability and impairing its target binding activity [27,28]. However, in rice, OsMPK3 exerts a positive regulatory role in enhancing cold resistance by impeding the degradation of OsICE1 during periods of cold stress [29].

Maize (*Zea mays*) is globally recognized as one of the most pivotal crops [30,31,32], with seed germination and seedling growth being particularly susceptible to cold stress, especially during the early spring season [33,34,35,36]. The growth of maize leaves and seedlings was adversely affected by the reduced rate of cell division caused by low temperatures [37]. When the temperature drops below −2.2 °C for a few minutes and remains below 0 °C for more than 4 h, the stems, leaves, and ears suffer irreparable damage [34]. The formation rate of leaves is decelerated by low temperatures, leading to a reduction in the overall leaf count [38]. Moreover, the growth of maize roots is also influenced by lower temperatures [39]. Consequently, exposure to lower temperatures can result in impaired germination, inhibited seedling growth, and even tissue or whole plant mortality, thereby potentially leading to crop failure [33,40,41]. Currently, several key genes involved in the response of maize to cold stress have been identified, such as *RR1* (*Response regulator 1*), *CesA* (*Cellulose synthase*), *MPK8*, *bZIP68* (*basic leucine zipper 68*), *bZIP113*, *TSAH1* (*Tryptophan synthase A homolog1*), *DREB1s*, and *ICE1* [42,43,44,45,46]. The identification of these genes holds significant implications for enhancing the cold tolerance of maize. However, the current comprehension regarding cold tolerance-related genes in maize remains incomplete. The exploration of more cold tolerance-related genes and regulatory mechanisms is of great significance for the development of cold-tolerant maize varieties. The MOR signaling network is necessary for normal growth and development of eukaryotes, potentially serving as a pivotal regulator in plant stem cell maintenance and cell polarization [1]. Furthermore, the MOR signaling network genes of *Arabidopsis* exhibit predominant expression in the shoot apical meristem and inflorescence meristem [1], and these developmental stages of maize are susceptible to cold stress [33,34,35]. The MOR signaling network genes potentially participate in the regulation of cold stress response in maize.

In this study, the genes comprising the MOR signaling network in maize were identified through sequence similarity searches using known components of *Arabidopsis*. The co-expression analysis was employed to ascertain the potential involvement of MOR signaling network genes in the regulation of cold stress in maize. Moreover, utilizing qRT-PCR (quantitative real-time polymerase chain reaction) analysis, it was ascertained that cold stress exerts regulatory effects on the expression of genes within the MOR signaling network. Subsequently, a mutant of one of these genes was identified and analyzed, thus elucidating its pivotal role in governing cold tolerance in maize. The results of this study will contribute to the elucidation of the regulatory mechanism underlying cold tolerance in maize and provide valuable gene resources for the breeding of cold-tolerant maize varieties.

## 2. Results

### 2.1. Identification of Pivotal Components of Maize MOR Signaling Network

**Figure 1:**
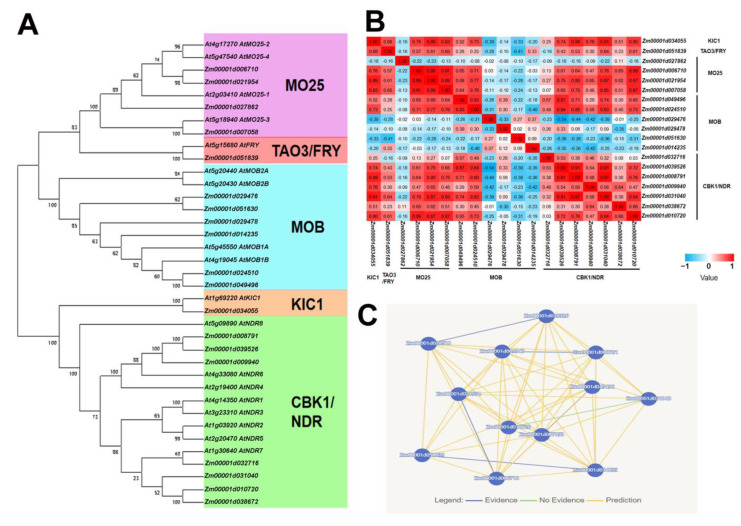
Identification of MOR signaling network genes in maize. (**A**) Phylogenetic analysis of MOR signaling network genes in maize and *Arabidopsis*. A total of 19 maize MOR signaling network genes were identified. The neighbor-joining algorithm is employed for the assessment of evolutionary distances. The numbers at the nodes represent the percentage of 1000 bootstraps. (**B**) Co-expression analysis of 19 maize MOR signaling network genes. In total. 12 of the genes had stronger correlations. (**C**) These 12 genes potentially interact with each other. The network map was generated using the Pathway Mapping function available on the MaizeNetome website (http://minteractome.ncpgr.cn./, accessed on 15 August 2023).

The *Arabidopsis* MOR signaling network genes were employed to query the maize genome annotation database using BLASTp, resulting in the identification of a total of 19 maize MOR signaling network genes. Among these genes, only one exhibits homology to *Arabidopsis* KIC1 kinase, another one shows homology to TAO3/FRY protein, four share homology with MO25 protein, six exhibit homology with MOB protein, and seven show homology with CBK1 protein (Figure 1A; Table S1). These 19 genes together constitute the MOR signaling network of maize, which may play a crucial role in growth and development. To further ascertain whether the identified MOR pathway genes belong to the same signaling network, we conducted a co-expression analysis among these genes. The KIC1 coding gene *Zm00001d034055*; TAO3/FRY coding gene *Zm00001d051839*; MO25 coding genes *Zm00001d006710*, *Zm00001d021954*, and *Zm00001d007058*; MOB coding genes *Zm00001d049496* and *Zm00001d024510*; as well as CBK1 coding genes *Zm00001d039526*, *Zm00001d008791*, *Zm00001d009940*, *Zm00001d031040*, and *Zm00001d010720* exhibit significant co-expression with each other (Figure 1B; Table S2). The results of co-expression analysis unveiled a robust interrelationship among these 12 genes out of the 19 MOR signaling network genes identified in this study, implying their potential collective regulation on the growth and development of maize. Furthermore, the interactions among these 12 genes were analyzed utilizing the Pathway Mapping function provided by the MaizeNetome website (http://minteractome.ncpgr.cn/, accessed on 15 August 2023) [47], unveiling potential interconnections and implying their participation in a common regulatory pathway (Figure 1C). Therefore, we have chosen these 12 genes as essential components of the maize MOR signaling network for further examination.

### 2.2. Co-Expression Analysis of MOR Signaling Network Genes and Cold Tolerance-Related Genes Revealed the Potential Regulatory Mechanism of Cold Tolerance in Maize

**Figure 2:**
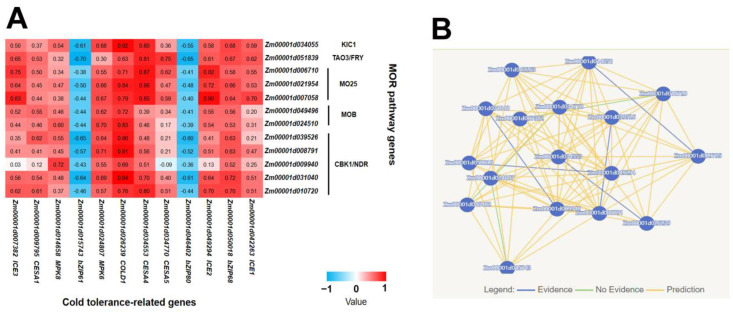
The MOR signaling network potentially participates in the regulation of cold tolerance in maize. (**A**) MOR signaling network genes and cold tolerance-related genes were significantly co-expressed. (**B**) There are potential interactions between CBK1 and genes related to cold tolerance. The network map was generated using the Pathway Mapping function available on the MaizeNetome website (http://minteractome.ncpgr.cn/, accessed on 15 August 2023).

To elucidate the contribution of MOR signaling network genes to cold stress tolerance in maize, we conducted a co-expression analysis between MOR signaling network genes and cold tolerance-related genes. The findings revealed a significant co-expression between genes involved in the MOR signaling network and multiple cold tolerance-related genes (Figure 2A; Table S3). All MOR signaling network genes showed significant co-expression with *COLD1*, a pivotal gene involved in the perception of cold stimuli [18]. These findings suggested that upon detection of a cold stimulus, COLD1 may directly transmit a signal of cold stress to genes involved in the MOR signaling network. Furthermore, eight, eight, seven, and six MOR signaling network genes were significantly co-expressed with *bZIP68*, *MPK6*, *CESA4*, and *ICE2*, respectively (Figure 2A; Table S3). These genes have previously been identified as pivotal regulators of cold tolerance in maize and *Arabidopsis* [24,44,45,46]. These results indicated that the MOR signaling network may be involved in the regulation of cold tolerance of maize. Moreover, we used the Pathway Mapping function of the MaizeNetome website (http://minteractome.ncpgr.cn/, accessed on 15 August 2023) [47] to analyze the interaction between cold tolerance-related genes and *CBK1* genes, as CBK1 potentially belongs to the MOR signaling network member that directly governs cold tolerance-related genes. The potential interaction between CBK1 and these cold tolerance-related genes further suggests the involvement of the MOR pathway in regulating cold tolerance (Figure 2B).

### 2.3. The Expression of Certain MOR Signaling Network Genes in Maize Was Regulated by Cold Stress

**Figure 3:**
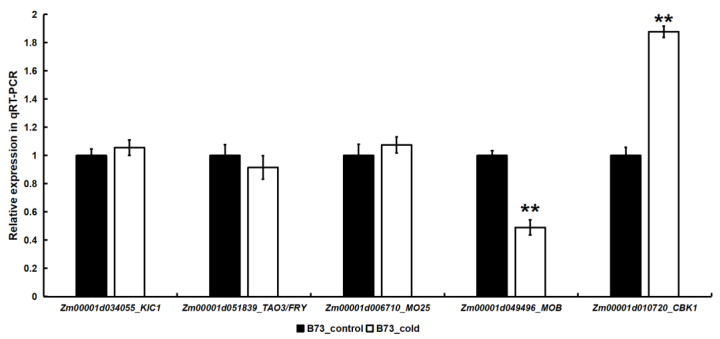
Cold stimulation regulated the expression of certain genes in maize in the MOR signaling network. ** significant at *p* < 0.01 by the Student’s *t* test.

We subjected maize B73 seedlings to cold treatment and assessed the expression of *Zm00001d034055*, *Zm00001d051839*, *Zm00001d006710*, *Zm00001d049496*, and *Zm00001d010720*, following cold stress using qRT-PCR. These five genes encode KIC1, TAO3/FRY, MO25, MOB, and CBK1, respectively, and represent key components of the MOR pathway. The results of gene expression analysis revealed a significant up-regulation in the expression of *Zm00001d010720* following cold treatment, as compared to the control, while there was a significant down-regulation in the expression level of Zm00001d049496 (Figure 3). Furthermore, the expression levels of *Zm00001d034055*, *Zm00001d051839*, and *Zm00001d006710* did not show any significant variation following cold treatment compared to the control (Figure 3). The findings suggested that the expression of some MOR signaling network genes was regulated by cold stress, potentially contributing to the modulation of cold tolerance in maize.

### 2.4. The Mutant of zm00001d010720 Exhibited Heightened Susceptibility to Cold Stress

**Figure 4:**
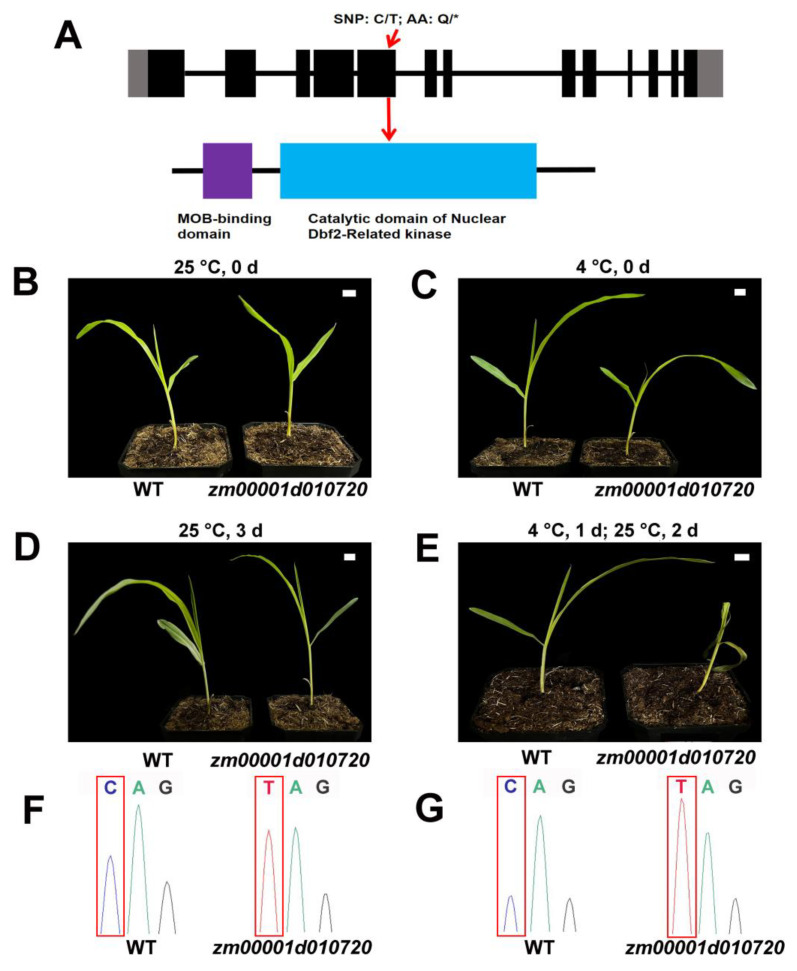
The mutant *zm00001d010720* showed increased susceptibility to cold stress. (**A**) Schematic diagram of *Zm00001d010720* gene with indicated mutation sites (**top**) and protein conserved domains (**bottom**). Black boxes represent coding regions, gray boxes represent the 5′ and 3′ untranslated regions, and lines represent introns. SNP, single-nucleotide polymorphism; AA, amino acids; *, stop gained. (**B**) Before cold treatment, the phenotypes of wild type and mutant seedlings in control group. Scale bar, 1 cm. (**C**) Before cold treatment, the phenotypes of wild type and mutant seedlings in cold stress group. Scale bar, 1 cm. (**D**) The phenotype of wild type and mutant seedlings in control group after 3 days. Scale bar, 1 cm. (**E**) The phenotypes of wild type and mutant seedlings after treatment at 4 °C for one day and recovery at 25 °C for two days. The mutant seedling displayed wilting and subsequent mortality. Scale bar, 1 cm. (**F**) Mutation site analysis of wild type and mutant seedlings in control group. (**G**) Mutation site analysis of wild type and mutant seedlings in cold stress group.

We further identified and analyzed a mutant *zm00001d010720*, which encodes the CBK1 kinase. The mutation of *Zm00001d010720* is a C-to-T substitution in exon 5, which results in the transformation of a glutamine residue to a premature stop codon, resulting in the partial deletion of the catalytic domain of kinase in Zm00001d010720, potentially impacting its protein functionality (Figure 4A). Fourteen-day-old WT (wild type) and *zm00001d010720* seedlings were subjected to a 24-h cold treatment at 4 °C and then we observed the growth of the plants after two days of recovery at 25 °C. After cold treatment, the *zm00001d010720* seedling exhibited wilting and subsequent mortality, whereas the WT seedling displayed normal growth (Figure 4C,E,G). However, both the WT and *zm00001d010720* seedlings without cold treatment could grow normally in the same period (Figure 4B,D,F). The results demonstrated that the mutation in the *Zm00001d010720* gene, which encodes CBK1 kinase, significantly impaired the cold tolerance of maize. This finding suggested that this gene plays a regulatory role in modulating the response of maize to low-temperature stress.

## 3. Discussion

### 3.1. Relevant Components of the MOR Signaling Network Exhibit Conservation in Maize

In this study, we identified a total of 19 MOR signaling network genes in maize, 12 of which were more closely related to each other and they may collaborate to govern the growth and development of maize, suggesting the conservation of MOR signaling network components in maize (Figure 1; Tables S1 and S2). The MOR signaling pathway was first discovered in yeast and is necessary for normal growth and development [3,4,5,6,7,8,9]. This signaling pathway has received limited attention in plants and has only been reported in *Arabidopsis* [1,2]. The MOR signaling network genes of *Arabidopsis* are mainly expressed as the shoot apical meristem and inflorescence meristem, which may play a crucial regulatory role in plant stem cell maintenance and cell polarization [1]. Currently, the elucidation of this signaling pathway in maize and its regulatory mechanisms governing plant cold tolerance remains elusive. However, it is expected that the expression pattern of these genes in maize will be similar to that observed in *Arabidopsis*. Additionally, at these developmental stages, maize is susceptible to cold stress hazards [33,34,35] and MOR signaling network genes possess the potential to participate in the regulation of the maize cold stress response. Furthermore, the functional characterization of these 12 MOR signaling network genes in maize remains unreported. However, the function of its homologous genes in *Arabidopsis* has been partially documented and these genes in maize may also exhibit similar functions to their counterparts in *Arabidopsis*. The *Arabidopsis* KIC1 protein is implicated in the regulation of cellular polarity, cell proliferation, cell expansion, and antibacterial immune responses [48,49,50]. The *Arabidopsis* TAO3/FRY protein is potentially implicated in the G-protein signaling pathway and exerts an influence on morphogenesis [51]. Furthermore, the *Arabidopsis* MOB protein interacts with KIC1 and CBK1 and is involved in the regulation of cell proliferation, cell expansion, pollen development and germination, and plant growth and development [48,52,53]. And, the *Arabidopsis* CBK1 protein plays a pivotal role in various aspects of growth and development, including embryogenesis, pollen development, and germination [53,54,55]. The findings of these studies suggested that genes involved in the MOR signaling network may also play a vital role in regulating maize growth and development.

### 3.2. The MOR Signaling Network Genes Play a Crucial Role in the Regulation of Cold Tolerance in Maize

**Figure 5:**
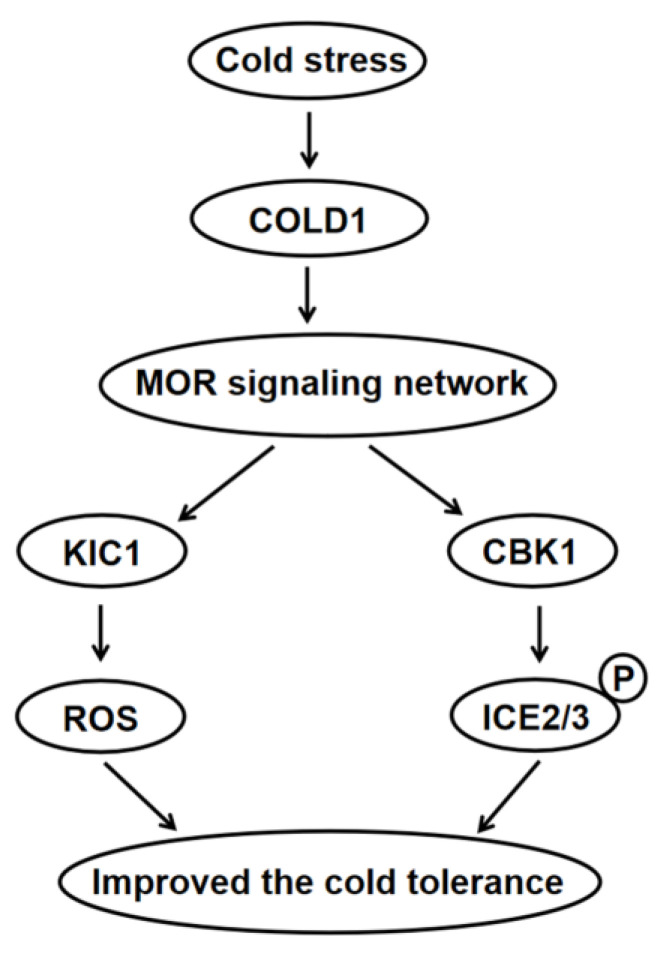
A putative model of the MOR signaling network regulating cold tolerance of maize. After being sensed by COLD1, the cold signal is transmitted to the MOR signaling network, potentially facilitating KIC1-mediated ROS generation, and phosphorylation of ICE2 and ICE3 via CBK1, thus enhancing the cold tolerance of maize. CBK1, Cell wall biosynthesis kinase; COLD1, Chilling tolerance divergence1; ICE2/3, Inducer of C-repeat binding factor expression2/3; KIC1, Kinase that interacts with cell division cycle1; MOR, Morphogenesis-related NDR kinase; ROS, reactive oxygen species.

Our study revealed significant co-expression between genes related to the MOR signaling network and multiple genes associated with cold tolerance in maize, with all MOR signaling network genes exhibiting a close relationship to *COLD1*, a pivotal gene involved in perceiving cold stimuli (Figure 2A; Table S3). After perceiving the cold stimulus, maize COLD1 may transmit a signal to the MOR signaling network genes, thereby triggering a cold tolerance response that is dependent on this specific signaling cascade. *COLD1* encodes the G-protein regulator [18,19,21], and the G-protein signaling pathway represents a crucial mechanism for transducing extracellular signals into intracellular responses [51]. The *Arabidopsis* TAO3/FRY protein exhibits a close association with the G-proteins [51], suggesting that COLD1 may potentially mediate the transmission of cold signals via TAO3/FRY. Through its interaction with KIC1 and CBK1, TAO3/FRY facilitates the phosphorylation of CBK1 by KIC1, thereby activating the MOR pathway to regulate growth, development, and stress response [9,12,15]. KIC1 can induce extracellular ROS (reactive oxygen species) burst, thereby positively modulating immune responses in *Arabidopsis* [49]. Furthermore, ROS also plays a crucial role in the regulation of cold stress response [20]. These results suggested that KIC1 may play a role in the regulation of cold stress through the modulation of ROS. In this study, the expression levels of some MOR pathway genes were modulated in response to cold stress, exhibiting a significant up-regulation in *Zm00001d010720* and a notable down-regulation in *Zm00001d049496* (Figure 3), indicating that cold stress regulated the MOR signaling network. These two genes encode the CBK1 kinase and MOB protein, respectively, which play a crucial role in regulating plant growth and development [48,52,53,54,55] and may modulate the cold tolerance of maize during the seedling. Our further study found that the mutation of *Zm00001d010720* rendered maize more susceptible to cold stress (Figure 4). This observation indicated that the MOR signaling network genes exert a positive regulatory influence on cold stress in maize. The mutation site of Zm00001d010720 is situated at amino acid position 302 within the protein-coding sequence, resulting in premature termination of the protein sequence at this site and subsequent deletion of several crucial protein binding sites, such as active sites and polypeptide substrate binding sites (Figure S1). In the future, we can identify proteins that interact with this protein of the WT and *zm00001d010720* and subsequently compare their differences to identify key downstream proteins associated with cold tolerance. Furthermore, we also analyzed upstream transcription factors that may regulate the expression of *Zm00001d010720*. Through co-expression analysis, we identified 252 transcription factors that exhibited co-expressions with *Zm00001d010720* (Table S4), further using the maize gene expression data from qTeller (https://qteller.maizegdb.org/, accessed on 13 August 2023). In total, 39 of these transcription factors were identified as having at least twofold changes in expression after cold stress, and these 39 transcription factors were more likely to act as regulatory genes upstream of *Zm00001d010720* (Table S4). We also used the plant promoter analysis website Plantpan4.0 (http://plantpan.itps.ncku.edu.tw/plantpan4/index.html, accessed on 13 August 2023) to analyze the promoter region of *Zm00001d010720* and identified that 8 of these 39 transcription factors may have binding sites in the promoter region of *Zm00001d010720* (Figure S2; Table S4). The presence of *bZIP68* (*Zm00001d050018*), a well-documented key gene associated with maize cold tolerance [46], among these eight identified transcription factors suggests that *Zm00001d010720* plays a pivotal role in the regulatory mechanisms governing cold tolerance in maize. In conjunction with the above studies, we postulate that the cold signal is transmitted to the MOR signaling pathway after being sensed by COLD1, potentially facilitating both KIC1-mediated ROS generation and phosphorylation of ICE2, ICE3, and other proteins via CBK1 to enhance the cold tolerance of maize (Figure 5).

## 4. Materials and Methods

### 4.1. Plant Materials

The materials used in this study included maize inbred line B73 (stored within our laboratory) and an ethylmethane sulfonate (EMS)-mutagenized stop-gained mutant (EMS4-0ab1d7, *zm00001d010720*) which were obtained from the Maize EMS-induced Mutant Database (MEMD; https://elabcaas.cn/memd/public/index.html#/, accessed on 20 August 2023) [56].

### 4.2. Identification of MOR Signaling Network Genes in Maize

The maize genome dataset downloaded from maizeGDB (Maize Genetics and Genomics Database, https://www.maizegdb.org/, accessed on 13 August 2023) was searched using BLASTp to identify the MOR signaling network genes, with the query sequences derived from the MOR signaling network genes in *Arabidopsis* [1].

### 4.3. Phylogenetic Analysis

The protein sequences of maize MOR signaling network genes were downloaded from maizeGDB, and the protein sequence of *Arabidopsis* MOR signaling network genes was downloaded from NCBI (National Center for Biotechnology Information, https://www.ncbi.nlm.nih.gov/, accessed on 13 August 2023). Protein sequences were aligned using MUSCLE in the MEGA5.1 software (v.5.1.1). The neighbor-joining algorithm was employed to assess evolutionary distances, while phylogeny testing was performed using the bootstrap method with 1000 replicates.

### 4.4. Co-Expression Analysis

The maize gene expression data from qTeller (https://qteller.maizegdb.org/, accessed on 13 August 2023) was utilized to perform a co-expression analysis of MOR signaling network genes with each other, as well as the co-expression analysis between MOR signaling network genes and cold tolerance-related genes (*COLD1*, *ICE1*, *ICE2*, *ICE3*, *MPK6*, *MPK8*, *bZIP61*, *bZIP68*, *bZIP80*, *CesA1*, *CesA4*, and *CesA5*) using SPSS software (v.24.0). Given the direct manifestation of leaf damage in maize following cold stress [44,45,46], we extracted 24 leaf-related expression data from the qTeller for co-expression analysis (Table S5). Significant co-expression was observed when the Pearson’s correlation coefficient was ≥0.60 [57].

### 4.5. RNA Extraction and qRT-PCR Analysis

The seeds of B73 were sown in pots and grown under controlled conditions at a temperature of 25 °C with a photoperiod of 16 h light and 8 h darkness. When the seedlings were fourteen days old, they were subjected to a cold stress treatment at 4 °C for a duration of 24 h. B73 cold-treated leaves and control leaves were taken, respectively, and 3 biological replicates were collected. All samples were immediately frozen in liquid nitrogen and stored at −80 °C. The total RNA from each sample was extracted using Trizol reagent (Invitrogen, Carlsbad, CA, USA). The expression of *Zm00001d034055*, *Zm00001d051839*, *Zm00001d006710*, *Zm00001d049496*, and *Zm00001d010720* in cold stressed B73 and control was validated using qRT-PCR. The Fast Quant RT Kit (TianGen, Beijing, China) was used to synthesize the first strand cDNAs. The qRT-PCR was then conducted using the Bio-Rad iQ5 (Bio-Rad, Hercules, CA, USA) according to the SuperReal PreMix Plus (SYBR Green) instructions (TianGen, Beijing, China). All reactions were performed with three technical replicates, and the expression levels were normalized using *GAPDH* (*Glyceraldehyde-3-phosphate dehydrogenase*) as an internal reference. The qRT-PCR primers are listed in Table S6.

### 4.6. Identification and Analysis of Mutant zm00001d010720

An EMS-mutagenized stop-gained mutant (EMS4-0ab1d7) of *Zm00001d010720* was obtained from MEMD (https://elabcaas.cn/memd/public/index.html#/, accessed on 20 August 2023) [56]. The material was grown at the Chongzhou Modern Agricultural Research and Development Base, Sichuan Agricultural University. The row length was 3 m, and the row width was 0.6 m with a plant spacing of 0.3 m within rows. The genotypes of each plant were determined using PCR, and the homozygous mutant seeds were obtained through self-pollination. The primers are listed in Table S6. The homozygous seeds of *zm00001d010720* and B73 were sown in pots and cultivated under controlled conditions at a temperature of 25 °C, with a photoperiod consisting of 16 h of light followed by 8 h of darkness. The fourteen-day-old seedlings were treated with cold stress at 4 °C for 24 h. After cold stress, the plants recovered at 25 °C for 2 days, and then the growth was observed.

## 5. Conclusions

In summary, we have successfully identified 19 maize MOR signaling network genes for the first time and observed that 12 of them exhibit a higher degree of co-expression, suggesting the conservation of these signaling network components in maize and their potential significance in maize growth and development. The subsequent analysis revealed the involvement of MOR signaling network genes in the regulation of cold tolerance. The expression levels of some MOR pathway genes were regulated under cold stress, and the mutation in *Zm00001d010720*, which codes CBK1, confers increased cold stress sensitivity upon maize. These findings suggested that genes associated with the MOR signaling network play a pivotal role in modulating cold tolerance in maize. This study has contributed novel genetic resources to facilitate the development of cold-tolerant maize varieties.

## Data Availability

Data is contained within the article and Supplementary Materials.

## Supplementary Materials

The following supporting information can be downloaded at: https://www.mdpi.com/article/10.3390/plants12203639/s1, Table S1: List of 19 MOR signaling network genes in maize; Table S2: Co-expression analysis of 19 maize MOR signaling network genes; Table S3: Co-expression analysis of MOR signaling network genes and cold tolerance-related genes; Table S4: Upstream transcription factors analysis of Zm00001d010720; Table S5: Expression data for co-expression analysis in this study; Table S6: Primers used in this study; Figure S1: Analysis of protein conserved domain of Zm00001d010720; Figure S2: Promoter analysis of *Zm00001d010720*.
