# Supplementary material for: Identification of Morphogenesis-Related NDR Kinase Signaling Network and Its Regulation on Cold Tolerance in Maize
Source: Plants (Basel). 2023 Oct 21;12(20):3639. doi: 10.3390/plants12203639 (PMC10610150; doi:10.3390/plants12203639)
Supplement: Supplementary file 1 [file plants-12-03639-s001.zip › Supplementary Figure S1.pdf]

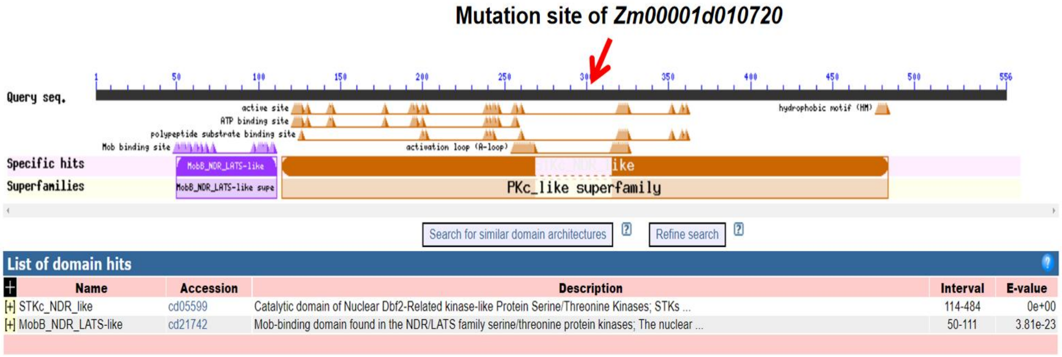

Figure S1 Analysis of protein conserved domain of Zm00001d010720. The mutation of Zm00001d010720 results in the deletion of several crucial protein binding sites, including the active site and the polypeptide substrate binding sites.
