# Supplementary material for: Identification of Morphogenesis-Related NDR Kinase Signaling Network and Its Regulation on Cold Tolerance in Maize
Source: Plants (Basel). 2023 Oct 21;12(20):3639. doi: 10.3390/plants12203639 (PMC10610150; doi:10.3390/plants12203639)
Supplement: Supplementary file 1 [file plants-12-03639-s001.zip › Supplementary Figure S2.pdf]

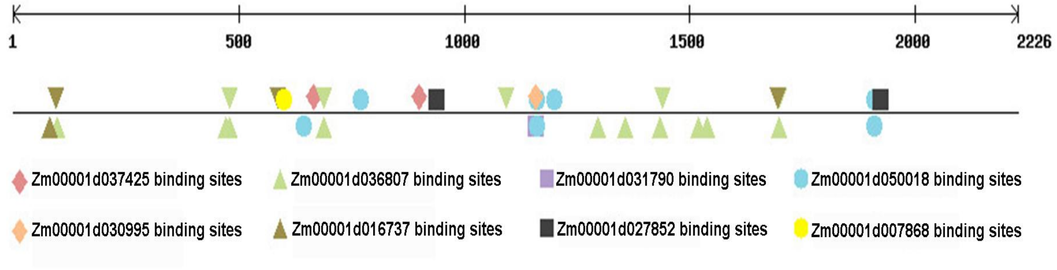

Figure S2 Promoter analysis of *Zm00001d010720*. In the promoter region of *Zm00001d010720*, binding sites of 8 transcription factors, including bZIP68, are found.
